# Blue Light Disinfection in Hospital Infection Control: Advantages, Drawbacks, and Pitfalls

**DOI:** 10.3390/antibiotics8020058

**Published:** 2019-05-07

**Authors:** João Cabral, Rodrigues AG

**Affiliations:** 1 Division of Microbiology, Department of Pathology, Porto Faculty of Medicine, University of Porto, 4200-319 Porto, Portugal; agr@med.up.pt; 2 CINTESIS—Center for Health Technology and Services Research, 4200-450 Porto, Portugal

**Keywords:** hospital acquired infection, multi-drug resistant organisms, infection control, photodynamic therapy, photosensitizer, porphyrins, blue light therapy

## Abstract

Hospital acquired infections (HAIs) are a serious problem that potentially affects millions of patients whenever in contact with hospital settings. Worsening the panorama is the emergence of antimicrobial resistance by most microorganisms implicated in HAIs. Therefore, the improvement of the actual surveillance methods and the discovery of alternative approaches with novel modes of action is vital to overcome the threats created by the emergence of such resistances. Light therapy modalities represent a viable and effective alternative to the conventional antimicrobial treatment and can be preponderant in the control of HAIs, even against multidrug resistant organisms (MDROs). This review will initially focus on the actual state of HAIs and MDROs and which methods are currently available to fight them, which is followed by the exploration of antimicrobial photodynamic therapy (aPDT) and antimicrobial blue light therapy (aBLT) as alternative approaches to control microorganisms involved in HAIs. The advantages and drawbacks of BLT relatively to aPDT and conventional antimicrobial drugs as well as its potential applications to destroy microorganisms in the healthcare setting will also be discussed.

## 1. Introduction

At present, hospital acquired infections (HAIs) have become a major problem responsible for millions of deaths and huge costs for health systems, especially if the causing agent is a multi-drug resistant organism (MDRO). Since most HAIs are potentially preventable, it is urgent to find alternatives to overcome this problem [1]. Due to the advances of science and technology, innovative ways to combat HAIs are arising, which can drastically reduce morbidity and mortality. 

There has been an enormous advance in the variety of antimicrobial agents available over recent decades, which provided clinicians with a previously unavailable wide array of therapeutic options. However, given the increasing use of these drugs, especially in hospital settings, the development of microbial resistance has become an emerging problem that represents tremendous costs and is responsible for very high morbidity and mortality. Microorganisms, such as bacteria, fungi, viruses, and parasites, can replicate very rapidly and acquire genetic traits, capable of promoting their survival in the presence of an antimicrobial agent. Such an organism can quickly become predominant among a microbial population, by positive selection. In addition to a wide array of mechanisms adopted by microorganisms to defend themselves against external aggression, many distinct factors may also contribute to the constant growth of antimicrobial resistance [2]. Examples of such factors are: 1. the inadequate or excessive prescription of antimicrobials, 2. the overcrowding of patients and understaffing in hospitals, 3. the generalized use of antimicrobials in the agro-food sector, 4. the facilitated transmission of microorganisms among populations, animal, and/or human, and 5. the increasing globalization and the expansion of poverty, mostly among third world countries.

Drug resistance is extremely costly not only for health services, but also for the patient, who is unable to obtain the maximum therapeutic benefit, and, for the society, where resistant microorganisms can spread. It was estimated that, if nothing is done in the meantime, by 2050, MDRO will kill 10 million people every year, which is a figure that will outweigh the death caused by cancer. According to this assumption, the cost of MDRO in terms of lost global production between 2015 and 2050 would be 100 trillion USD, if no action is to be taken [3].

HAIs caused by MDROs represent a serious health problem. In fact, not only the treatment of infectious diseases would be affected, but also several common clinical procedures such as cesarean sections, organ transplants, and chemotherapy, that strictly depend on the use of antibiotics to prevent infections may be at risk [4]. Therefore, there is an urgent need to develop innovative and effective measures to combat pathogenic resistant microorganisms, which is often refractory to conventional treatment, as well as limiting the development and spread of antimicrobial resistant microorganisms, particularly at niches/reservoirs like walls, floors, and other hospital surfaces.

Further aggravating the panorama, pharmaceutical companies are withdrawing from the market. In fact, since the development of new antimicrobial agents is highly costly and resistances can arise in a few years, companies cannot profit as much as they used to and have focused in other more profitable markets [5].

Alternative approaches to avoid the progression of HAIs must be pursued since millions of human lives are at risk. Furthermore, the prevention of HAIs and MDROs should be focused on non-antibiotic methods.

Antimicrobial blue light therapy (aBLT), which is a specific type of antimicrobial photodynamic therapy (aPDT), is, at present, attracting increasing attention due to its intrinsic antimicrobial effect without the addition of exogenous photosensitizers (PS) [6,7]. The use of aBLT reduces the possibility of potential harmful effects on eukaryotic cells, which reduces the possibility of human tissue damage. Moreover, the aBLT impact in non-pathogenic microorganisms may be somewhat reduced when compared with that of aPDT. Given these characteristics, there is an increasing interest in exploring the potential application of aBLT in the sanitization of healthcare facilities, such as in the disinfection of hospital wards, patient rooms, and operating theaters with patients admitted. High Intensity Narrow Spectrum light (HINS), which takes advantage of a wave length of 405 nm, can be used by installing special lighting systems in the selected room. This type of sanitization can be more effective when compared to the usual manual cleaning, which, by being highly worker-dependent, has a lot of variability [8]. 

Despite not being addressed in this review, another innovative disinfection technology worth mentioning is Ultraviolet C light (UVC) emitted by Light Emitting Diodes (LEDs). In fact, it was proven to be effective in the disinfection of various surfaces, including medical devices, without significantly damaging them. In a study recently performed, UVC LED were able to successfully disinfect a stethoscope. The implementation of this technology could contribute to an effective, safe, and cheap disinfection method of various hospital surfaces over the long term [9].

## 2. Methods

To prepare this review, research was made regarding information present in scientific articles collected via PubMed, via a Web of Knowledge, and via Scopus.

The main keywords used were phototherapy, photosensitizing agents, microorganisms, hospital acquired infections, drug resistance, and disinfection.

Primary selection of the most appropriate articles was conducted by title and abstract evaluation and secondary selection was made after careful reading of the previously selected articles, which confirms the adequacy of the information provided.

Further research was made by using secondary terms and other search resources with the aim to uncover additional bibliography items of potential interest for this topic.

## 3. Hospital Acquired-Infections

### 3.1. Reservoirs and Transmission

Hospitals are, on a daily basis, confronted with multiple patients, possibly infected with pathogenic microorganisms, often MDRO, which represents a major challenge in terms of maintaining its facilities secured and disinfected.

The reservoirs of such pathogens not only involve medical indwelling devices (MIDs) (such as intravenous catheters), but also many distinct surfaces of the hospital [10]. The most common reservoirs involve patient’s own microbiota and the hands and nostrils of healthcare personnel [11]. However, it is well-known that contaminated surfaces also play a central role in the spreading of infections, like MRSA and Vancomycin Resistant *Enterococcus*, where they can survive for weeks in inorganic surfaces, if not submitted to a correct disinfection [12,13]. The most commonly infected surfaces are called High Touch Surfaces (HTS), which can be divided in patient care items, such as blood pressure cuffs, stethoscopes, and the thermometer as well as environmental surfaces, that are further divided in medical equipment (such as monitor touch screen, controls and cables, and supply carts) and patient rooms (bed rails/controls, bedside table/handles, chairs, telephone and TV remotes, light switches, door and closet knobs/plates, toilet seat, flush handles and bedpan cleaners, sinks and soap dispensers, and trash cans) [11]. Recent studies even demonstrated that physician’s mobile phones and boots are possible carriers of methicillin-resistant *Staphylococcus aureus* (MRSA) [14,15].

The transmission of pathogens among healthcare settings can occur in a wide variety of ways. Microorganisms can be transmitted from their source to a new host through direct or indirect contact, in the air and/or water, or by vectors with indirect transmission being the most common [16,17]. Transmission through vectors is more prevalent in tropical countries, where insects can transport the pathogens. Airborne transmission results from pathogen-laden droplets expelled from infected patients into the air when he sneezes, coughs, speaks, or simply breathes, but can also result from problems in hospital ventilation systems [18]. The same situation applies for hospital water systems that can be colonized by microorganisms and be a source for waterborne infections in which *Legionella* infections are a well-known example of these situations [19]. Transmission by direct contact between patients is more uncommon. Indirect contact, as previously mentioned, is the most common route of infection, which consists of a transfer by a healthcare worker contaminated with organisms from their own body, or from another patient, to the patient whom he is taking care of.

### 3.2. Challenges and Costs

There is increasing interest in a more comprehensive understanding of hospital-acquired infections and how to control them. Even though a lot of progress has been made, there are still many fields to improve and new challenges are always arising [20]. Not only patients’ lives are seriously at risk, but there are extremely high costs to the health care system in which HAIs are the most common complication of hospitalized patients [21]. HAIs lead to prolonged hospital stays, to the increase of microbial drug resistance, to a massive additional financial burden and, ultimately, to unacceptable deaths [22].

Despite all the efforts developed to combat these infections, the World Health Organization (WHO) estimates that the prevalence of HAIs in developed countries varies within the range of 3.5–12% and in developing countries within the range of 5.7–19.1%. Such infections are responsible for a minimum of 37,000 deaths annually in Europe. Among the 1.7 million HAIs estimated to occur annually in the USA, 99,000 result in death [22,23]. The most recent studies in Europe report a prevalence of 6.0% of patients with at least one HAI in acute care hospitals in 2011 [24].

In terms of costs, it is also a terrific scenario since WHO estimates a 7 billion euro loss in Europe each year related to HAIs. This is similar to the USA where the estimated value is $6.5 billion [22]. To better understand the magnitude of these numbers, the direct medical cost of preventable HAIs is comparable to the annual costs in the USA for stroke ($6.7 billion), diabetes mellitus with complications ($4.5 billion), and chronic obstructive lung disease ($4.2 billion) [25].

## 4. Prevention and Control of Hospital-Acquired Infections 

### 4.1. Surveillance Methods

The primary and most effective step toward the elimination of HAIs has always been its prevention. Ideally, the gold standard of surveillance would be a prospective, continuous, and on-site system that covers the whole hospital. Unfortunately, such a program rarely takes place. A surveillance method to be viable in hospitals must be financially equilibrated. This means that the costs saved by preventing HAIs through the method must outweigh its costs of implementation [26].

A study conducted in Austria in 2010 consisted of the introduction of a Nosocomial Infection Surveillance System (NISS), in two surgical and in one general intensive unit care (ICU), in order to improve safety and quality of treatment. After one year, it was demonstrated that the NISS was an effective method to improve patient’s safety, allowed the generation of data for comparative evaluation of infection rates, and was a helpful tool for the professionals, since it made it possible to better appreciate the risks of medical procedures and to learn from previous data [27,28].

It is important to notice that, according to the European Centre for Disease Prevention and Control (ECDC) data from the first European-wide point prevalence survey, of all patients admitted to European hospitals in 2011 to 2012, 6% are infected with at least one HAI [29]. In the clinical practice, at least 20% of HAIs may be preventable by sustained and multi-faceted infection prevention and control program, including surveillance of HAIs, which demonstrates the importance of the implementation of active policies [30].

### 4.2. Infection Control Programs

Due to the high morbidity and mortality of HAIs associated with the increasing emergence of MDRO, the implementation of infection control programs in all hospital facilities is crucial. This requires a multi-disciplinar and multi-professional approach, including all related stakeholders such as physicians from diverse specialties (epidemiologists, infectiologists, surgeons, anaesthesiologists, microbiologists, etc.), nurses, pharmacists, cleaning employees, architects, and hospital administrators.

WHO has been strongly warning that hands are the most common vehicle for transmission of organisms and “hand hygiene” is the single most effective mean of preventing the horizontal transmission of infections among hospital patients and healthcare personnel [31]. This also underlines the importance of standard contact precautions among healthcare workers and patients, reserving strict isolation measures to patients with highly transmissible and/or MDRO [32]. 

There are multiple approaches to avoid accumulation and transmission of hospital pathogens. The conventional method, most commonly used nowadays, is the manual cleaning with detergents, performed by housekeepers, which is worker-dependent and has specific concerns [8,33]. Although it is a cheaper technique in a short-term period, it can get much more expensive and put patients in risk in the long-term. In fact, manual routine cleaning techniques are often suboptimal. They are personnel dependent, which leads to diverse problems [34] due to the different nature of surfaces being cleaned. Some parts are left without proper cleaning, without cleaning 40% to 50% of surfaces that should be disinfected by the staff [35]. The disinfectants used can be inadequate for a certain kind of microorganism, can be contaminated, and can also have an inappropriate over-dilution [36]. This results in a quick return to elevated levels of pathogens after a few hours of cleaning, which is understandably ineffective.

Ventilation systems are widely used in hospitals. They can be natural or mechanical, being the last very commonly available in high/medium income countries. They help renovate the air, diluting the particles in it, which are able to reduce the infections by *Mycobacterium tuberculosis*. Not only tuberculosis is transmitted through the air, but also a wide and growing variety of other infectious organisms, which can travel in the air via droplets and infect other patients or surfaces if the ventilation is not effective [37]. It is very important to regularly monitor the functionality and microbiological quality of these systems in order to reduce the spread of airborne infections. The same consideration is valid for the healthcare facility water supply. Waterborne infections, including *Legionella spp*., may be prevented by implementing firm hygiene practices and it is important to constantly revise the water management programs [38].

### 4.3. Limitations of Current Methods and New Perspectives

The most commonly used methods for hospital cleaning, mostly involving manual routine cleaning techniques, are far from perfect. In fact, studies demonstrated that there is a significant potential to decrease 10% to 70% of HAIs, depending on the conditions of each case [30]. 

Clearly, there is not a method without any flaws, but, with the advances in science and technology, it is impossible to continue to ignore the potential of the new and innovative methods that are arising, which can lead to a more strict and effective prevention of HAIs. The ideal method should be highly germicidal, act continuously (since the contamination of hospital rooms is frequent [12]), non-harmful for humans, simple, and cost-effective.

New methods such as self-disinfecting surfaces, hydrogen peroxide vapor, steam cleaning, ultraviolet light devices, aPDT, aBLT and High Intensity Narrow Spectrum light (HINS) [8,39], are only a few in the vastness of methods that already exist and are available to use daily in the hospital setting.

The main objective is not to stop using the conventional techniques, but to allow new methods to be used, which can, thus, fill each other’ gaps and reach the best disinfection rates.

## 5. Photodynamic Therapy as an Alternative Approach in the Control of Colonization/Infection in Hospital Settings and Facilities

### 5.1. Antimicrobial Photodynamic Therapy (aPDT)

Due to the golden era of antibiotics, PDT was somewhat forgotten, but recently, with the unstoppable arising of MDROs, it was suggested as a promising solution for HAIs. PDT is extensively explored in the treatment of cancer, whereby studies on PS distribution, light exposure, light sources, and endoscopy equipment previously used for cancer treatment, can also be aPDT. In the treatment of cancer, the PS is usually injected into the bloodstream and accumulates in the tumor. However, to this day, aPDT has only been applied to localized infections rather than systemic. In the treatment of localized infections, the PS should be applied to the infected area by topical application, infiltration, injection, or aerosol [40]. When aPDT is intended to inactivate microorganisms on surfaces, such as in healthcare settings, its application is much easier.

#### 5.1.1. Mechanisms of Action (Type I and Type II)

The effect of photodynamic inactivation results from a series of photophysical and photochemical events, which result from the photosensitizer (PS) excitation by light, which, through two distinct pathways (mechanisms of action type I and type II), lead to the production of reactive oxygen species (ROS), which will oxidize the biomolecules [41].

**Figure 1:**
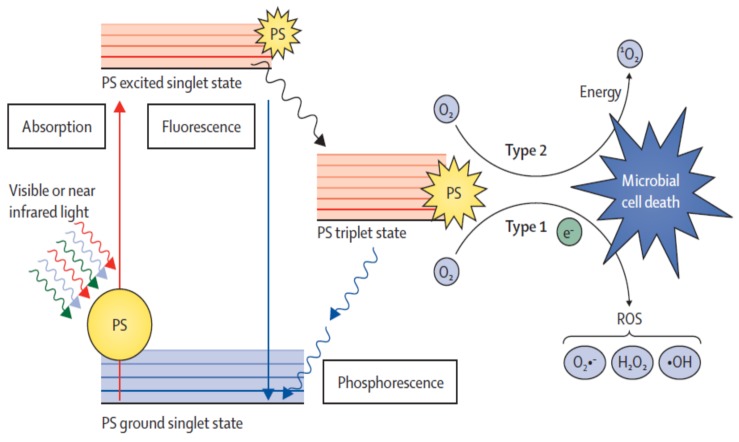
Jablonski diagram showing photodynamic mechanism and respective pathways (Type I and II) (adapted from Wainwright, 1998 [49]).

In the dark, the PS is in the electronic ground state configuration. The absorption of a photon by the PS at a given wavelength causes its excitation to a new electric state, which has a short lifetime (life time 10^−9^ to 10^−6^ s) [42]. The excited PS by emitting light returns to the ground state or undergo cross-system intersection and convert to an excited triplet state that has a superior lifetime (10^−3^ to 10 s) [42]. Once in this state, the PS has two paths to return to a basal state by spin inversion, with light emission (phosphorescence) or by a non-radiative mode. Given that the triplet state has a prolonged lifetime, free radicals of oxygens are generated by type I reaction, or transfer energy directly to molecular oxygen by type II reaction, which produces singlet oxygen (Figure 1). These reactions (type I and type II) give rise to lethal species, which causes irreparable oxidative damage in different vital cell targets [42,43,44].

The type I mechanism involves the abstract concept of the hydrogen atom or the transfer of electrons between the excited PS and a substrate, which obtains radical species (Table 1, Equations (1) and (2)). These free radical species interact with oxygen and create ROS, like the superoxide radical anion (Table 1, Equation (3)). The superoxide radical is not very reactive in biological systems, but, when protonated, can produce hydrogen peroxide and oxygen (Table 1, Equations (4) and (5)) or the extremely reactive hydroxyl radicals (Table 1, Equations (6)–(8)).

The type II mechanism is simpler, leading only to the production of singlet oxygen. In this way, the excited triplet state of the PS (3PS) the excess of energy is transferred to the molecular oxygen (^3^O_2_), returning to its basal state (1PS) and producing singlet oxygen (Table 1, Equation (9)). Like radical species, ^1^O_2_ is highly electrophilic and interacts with various biomolecules, which inactivates different microbes (Table 1, Equation (10)).

Both reactions originate ROS that instantly interact with biological components of the cell wall, like proteins, lipids, amino acid residues (cysteine, histidine, and tryptophan), nucleic acid bases (guanine and thymine), and the pigments in certain cells [45,46]. Due to the high reactivity and short half-life of ROS, only molecules and structures close to the production area of singlet oxygen and free radical species are directly affected and destroyed [47].

The two types of mechanisms may occur simultaneously or separately, the predominance of each depends on the used PS, the substrate, and the molecular oxygen concentration [42]. The predominant mechanism may be altered during the process [41,48,49].

#### 5.1.2. Photosensitizers

The PS has a special role in PDT, since it is responsible for generating ROS, after being light activated [50,51,52]. The capacity of the PS to absorb light at a certain wavelength relate to its structure and to the electronic absorption spectrum.

Most PS tested in aPDT are tetrapyrrol derivatives, known as porphyrins, even though some non-tetrapyrol derivatives have also been subject of the studies. Porphyrins intervene in diverse vital functions, namely the respiration (heme group) and photosynthesis (chlorophyll and bacteriochlorophyll). This class of PS was the first type of compounds to be used in PDT against tumors and to be allowed for clinical use (e.g., Photofrin^®^) [50,53]. Based on these macrocycles, several synthetic analogues have been developed such as meso-tetraarylporphyrins, phthalocyanines, porphycans, texaphyrins, and safirins, which exhibit very promising characteristics for use as PS [53]. 

According to the literature, a good PS should display several characteristics to be used in aPDT [54,55,56]: (1) high chemical purity and simple synthesis, (2) photostability, so that it can be used in aPDT without being quickly degraded, (3) solubility (the PS does not aggregate or precipitate. If the PS aggregates, it is no longer available to bind to microorganisms and, consequently, there is a decrease of its function), (4) positive charge, mainly for the inactivation of Gram-negative bacteria. Photoinactivation is more effective with positively charged PS because it promotes a tighter electrostatic interaction with the negative charges at the surface of bacteria, and (5) amphiphilic properties. Some studies showed that a PS with amphiphilic character exhibits more affinity to the microorganisms.

#### 5.1.3. Light Conditions

The characteristics of incident light, irradiance source, and total light dose assume a vital role in the performance of aPDT and must be taken in account for the development of effective protocols.

The experimental results show that for the same PS and microorganism, the photodynamic efficiency depends on the light source used. The photodynamic inactivation requires a light source to activate the PS (visible light or near the visible) at wavelengths where the PS absorbs it efficiently [53]. The light source for aPDT should exhibit suitable spectral characteristics, preferably coincident or close to the maximum absorption wavelength of the PS to promote a photodynamic effect by generating enough ROS.

In the inactivation of microorganisms, a wide variety of coherent and non-coherent light sources have been tested, ranging from the laser to tungsten filament lamps. In the aPDT, low power light is used and microbial inactivation can be achieved even with powers in the order of milliwatts [53].

The wavelength of light required for the induction of aPDT further depends on the structure and the absorption spectrum of the PS. For example, for porphyrin derivatives irradiated with white light, the efficacy of microbial therapy appears to decrease with increasing wavelength. For these PS, blue light (400 to 480 nm) is more effective at a microbial inactivation than green (480 to 550 nm) or red (600 to 700 nm) light. The blue light of shorter wavelength (400 to 450 nm) is more phototoxic compared to that of the blue light’s longer wavelength. Although red light is not as effective as blue light, it can be more effective for treating infections because it penetrates better into animal tissues [53].

The light dose used in aPDT also influences microbial inactivation in a time and irradiance-dependent manner. The few studies on the influence of light parameters currently available in the literature suggest that exposure to light with high irradiance in a short period of time can give different results, in terms of microbial inactivation than those obtained with exposure to low irradiance light over a long period of time, even if the total dose of light is the same. In general, the aPDT is most effective with a low irradiance light and a longer treatment time [57]. Gábor et al. also demonstrated a higher inactivation rate of *E. coli* and *E. hirae* when a total dose of white light (irradiance 0.08 to 0.25 W/cm^2^) was received for a longer period [58]. For a T4-type phage with a high light dose (216 J/cm^2^), the PDI rate was higher when lower radiances were used, namely 150, 300, and 600 W/cm^2^ [57]. 

Conventional lamps, also designated as non-coherent light sources, were the first to be used in PDT assays because they were cheap, accessible, and easy to use. However, they lack other features, like the ability to control the light dose applied. At present, to overcome such limitations, lasers, which are also known as coherent light sources, started to be used in aPDT. They become widely used due to their ability to produce a monochromatic light (with an exact wavelength) and to control a light dose [50,51,52,54,59]. One other important factor is to match the wavelength with the chosen PS in order to maximize the yield of produced ROS [51,52]. Regarding the influence of the tissue, it is important to mention that the travel direction of light is also affected by the inhomogeneity of the cells, namely the presence of organelles, macromolecules, and the interstitial layers in fungi.

For environmental applications, such as healthcare settings’ disinfection, the use of a wide wavelength potent light source in aPDT is a clever choice because it can be used for different PS, which makes the process cheaper and easy to implement.

#### 5.1.4. Microbial Targets

The process of aPDT is clearly highly dependent on its targets and it is essential to understand them to maximize the effectiveness of the process. Different studies state that the key target of aPDT is influenced by the proper chemical structure of the PS, by the target microorganism, and by the PS photoinactivation mechanism [53,60].

Despite the multi-target nature of aPDT, the major microbial targets are proteins and lipids of the outer structures of the microorganisms (e.g., cytoplasmic membrane, cell wall, capsid, and viral envelope) rather than nucleic acids.

Proteins: 

Cell membrane proteins of bacteria, fungi, protozoa, and virus capsid proteins are considered the main targets of photoinactivation, for their preponderant role in the cells, for their abundance, and for their easiness to bind to the porphyrins. The oxidation of proteins leads to the formation of protein peroxides and carbonyl compounds, formation of cross-links and aggregates, the changes of molecular conformation of proteins, and the inactivation of enzymes and loss of proteins [45,53,60].

Lipids: 

ROS can cause both direct and indirect oxidative modification of the lipids. Lipid hydroperoxides are intermediates in the peroxidation process, formed by its interaction with singlet oxygen, which can modify the affected cell components, being the oxidation extended to the surrounding environment [61,62].

Nucleic acids: 

Nucleic acids are not a preponderant target of photoinactivation, even though they can strongly bind to PS. This is due to the photodynamic inactivation being a multi-target process that mainly affects the external constituents of the microorganisms and, since the ROS are not generated in the nucleus, the limited lifespan and their outer location restricts its area of action.

### 5.2. In Vitro and Clinical Effectiveness of Photodynamic Therapy

Although aPDT is effective against bacteria, viruses, fungi, and parasites, its inactivation efficiency varies according to the microorganism. In general, bacteria and viruses are more easily inactivated than fungi and parasites. Spores of bacteria and fungi, particularly endospores, and parasite eggs and cysts are more resilient to inactivation than the corresponding vegetative cells [53].

Currently, there is no routine application of aPDT for treatment of microbial infections, apart from the use of dihematoporphyrin ether and delta-aminolevulinic acid in the treatment of skin infections by *Propionibacterium acnes*, local *papilloma virus* infections, and cutaneous *leishmaniosis*. 

Nonetheless, aPDT with methylene blue under visible light and psoralen and rivoflavin under UV light, are already approved for plasma disinfection in a few countries [63]. However, having to account for the effective microbial inactivation in these laboratorial assays as well as in the few clinical trials already conducted, other applications of aPDT can be forecasted, such as the disinfection of infected skin, wound treatment, oral cavity, and soft-tissue infections as well as treatment of abscesses and environmental disinfection.

Bacteria: 

Gram-positive bacteria are more easily inactivated than Gram-negative bacteria. The difference in the sensitivity of the two groups is related to their different cell wall composition. Most Gram-positive bacteria have a cell wall consisting of several layers of peptidoglycans, negatively charged, which exhibit a relatively high degree of porosity. Macromolecules having a molecular weight of 30,000-60,000 (e.g., glycopeptides and polysaccharides) can easily pass through this structure. Consequently, most PS can go through their membranes, since its molecular weight generally is situated between 1500 to 1800 Da [64]. On the contrary, Gram-negative bacteria display in the cell wall, an additional highly organized outer membrane, which are external to the peptidoglycan layer. The asymmetrical nature of the outer membrane is due to the distribution of its phospholipids, proteins, lipoproteins, and negatively charged lipopolysaccharides [65] which does not allow the passage of various molecules into its interior. However, hydrophilic molecules of 600–700 Da can diffuse through the porins [66].

Gram-positive bacteria can be efficiently inactivated by neutral and anionic PS since the diverse PS can effortlessly go through their highly permeable cell wall. Nevertheless, these PS are not effective against Gram-negative bacteria [67], unless they are co-administered with external membrane disrupting agents such as CaCl_2_, EDTA, and polymyxin B, which can lead to electrostatic repulsion and destabilize the cell wall [44,68]. Gram-negative bacteria can be directly and effectively inactivated by cationic PS since these PS are able to bind to the negatively charged components of the outer membrane and allow a more effective interaction [67]. 

According to the literature, there are also differences in susceptibility to aPDT within each of the two bacterial groups, which are Gram-positive [53,69] and Gram-negative [53,70,71]. These variances are also due the differences in the cell wall of each bacteria [69]. Gram-negative bacteria have variances in their layers of peptidoglycan and lipidic outer membranes. As for the typical Gram-positive bacteria, there is no significant difference in their structure/composition.

Viruses: 

In viruses, several studies suggest that lipid-enveloped ones are more susceptible to PDT [40,53,60]. It is also suggested that different types of nucleic acids viruses (DNA and RNA) present different susceptibility to PDT, but the differences between RNA and DNA phages are not only attributed to their nucleic acid type, but also to the composition of their capsids [41]

The clinical application of aPDT to inactivate viruses has been successful. Neutral red/proflavine was effectively used to treat herpesvirus genital infection without relevant side effects [72]. Porphyrins were shown to be effective against herpes virus, the influenza virus, and the Papillomavirus [73,74]. aPDT is already approved to sterilize plasma. Different viruses such as hepatitis viruses, parvoviruses, the West Nile virus, and HIV have been effectively inactivated by methylene blue [75,76].

Fungi and Parasites:

Unlike bacteria and viruses, fungi and parasites are compartmented cells and, consequently, whenever the cell wall and membranes are damaged by the ROS, the PS enter its interior. Similar to bacteria, fungi also have a cell wall, which is more permeable to external substances than Gram-negative bacteria wall, but less than Gram-positive [77]. Since ROS are highly reactive and have a short lifetime, the localization of the PS into the cell is very important, since the organelles located nearby to the PS have the highest probability of being affected. 

Since fungi and parasite cells are larger when compared to bacteria and viruses, the amount of ROS needed to kill such a larger cell is much higher than is necessary to kill a bacterial cell or a viral particle [78]. On the other hand, the eukaryotic cell structure makes aPDT effect more difficult to work for these microorganisms than for bacteria and viruses. However, effective inactivation of fungi and parasites has already been observed [79,80]. In fact, to attain the effective inactivation of fungi and parasites, it is necessary to adjust the aPDT conditions as well as increase the PS concentration and the light dose [81]. Notably, *Candida* species are effectively inactivated by aPDT, but they are not as susceptible to PDT as several prokaryotic bacteria, including *Staphylococcus aureus* or *Streptococcus mutans* [82].

Likewise, it was observed that aPDT is effective for inactivating parasites, but also requires a higher PS concentration and higher light doses than those required for bacteria and viruses. aPDT with different PS have been tested for the inactivation of *Leishmania sp*. [83,84] and *Plasmodium falciparum* [85]. Cysts of *Colpoda inflate* and eggs of helminths like *Ascaris lumbricoides* and *Taenia sp* were also successfully photo inactivated [86].

### 5.3. Effectiveness of Photodynamic Therapy in Healthcare Settings/Facilities 

The potentialities of aPDT to eliminate microorganisms, even MDROs, surpasses the treatment of human infections, with a particular focus on the healthcare facilities. High doses of light can be used to destroy microorganisms effectively on surfaces. Moreover, higher concentrations of PS can be applied, which can be supported in membranes/films. This allows its recovery and recycling, which makes this approach durable, sustainable, economic, and environmentally-friendly. In fact, recently, a lot of developments were performed to renew the way that aPDT and its PS can be used. New methods have been tested to allow the immobilization of PS in diverse supports, which permits its use in the disinfection of materials and surfaces [87,88].

According to the literature, photoactive compounds/materials can potentially be used to prevent/eliminate microbial colonization in healthcare settings. Among these materials, nanoparticles, silica, chitosan and cellulose biopolymers, liposomes, nanogels, and carbon photoactive compounds stand out, which seem to be promising for disinfection/sterilization of polymeric materials (sodium chloride bags and tubing, gloves catheters, syringes, and hemodialysis filters), protective clothes, masks and bedclothes, walls, floors, and instruments, as well as hand disinfection of healthcare providers and even air disinfection of healthcare facilities [87,88,89,90,91].

Disinfection/sterilization of walls, floors, instruments:

Healthcare surfaces are constantly contaminated with many microorganisms, including MDRO, which perpetuates the transmission of HAIs. Since surfaces are an important reservoir of pathogenic microorganisms, control of surface contamination with effective approaches such as aPDT can prevent its recontamination.

A surface coating of cellulose impregnated with toluidine blue O was contaminated with Gram-positive and Gram-negative microorganisms. After 24 h of irradiation with white light, a 4 and 5 log reduction of *S. aureus* and *E. coli*, respectively, was observed. The authors suggested that this material has the potential to be used as wall paint to reduce the spread of nosocomial infection [92].

### 5.4. Disinfection/Sterilization of Polymeric Materials

Antimicrobial materials based on polysiloxane (a polymer used in catheters) and incorporating methylene blue and gold nanoparticles were evaluated against *Escherichia coli* and MRSA). There was a reduction of *E. coli* by 1.0 log after 5 min of irradiation with a red light (660 nm, 250 mW, and of *MRSA* by 3.5 log reduction, without affecting the mechanical properties of the photoactive material [93,94].

Other photoactive materials, based on polymers (polyurethane, silicone) used in catheters and in hospital touch surfaces (screen protectors for telephones and tablets, covers, keyboards, and hand dryers), embedded with MB, toluidine blue O (TBO), crystal violet (CV), and gold nanoparticles were also effective to inactivate MRSA, *S*. *epidermidis*, *Saccharomyces cerevisiae*, *E*. *coli*, *bacteriophage MS2*, the fungus-like organism *Pythium ultimum*, and the filamentous fungus *Botrytis cinerea* [94,95,96,97,98,99,100,101].

In terms of disinfection/sterilization of protective clothes, dressings and bedclothes, another strategy to combat HAIs is the use of antimicrobial textile products, such as gowns, beddings, dressings, and bedclothes. A stable, durable, and washable fabric of cellulose coated with a layer with ε-polylysine and a second layer with a zinc phthalocyanine PS, exhibit a potent antimicrobial activity against both Gram-negative and Gram-positive bacteria. The survival of *E. coli* and *S. aureus* decreased by 99% and 98%, respectively, after aPDT with this photoactive material. Besides, this fabric was also effective to inactivate a drug resistant bacterial strain [102].

### 5.5. Advantages and Drawbacks of Photodynamic Therapy Relatively to Conventional Antimicrobials

aPDT shows many strengths when compared to conventional antimicrobial therapy [49,54,103] (1) such as multitarget and broad-spectrum activity. Unlike conventional antimicrobials, photo antimicrobials can inactivate bacteria (both Gram-positive and Gram-negative), viruses, fungi, and parasites, which can be useful to empiric treatments. (2). Less probability of resistance development: since aPDT is not specific and involves *in situ* production of ROS that can affect several biomolecular sites, this therapy bypasses the usual mechanisms of resistance. Unfortunately, microorganisms can easily develop resistance against many of the available conventional antimicrobials, due to their single mode of action, which constitutes a major advantage of aPDT compared to conventional antimicrobials [104,105,106]. (3). Effectiveness against MDR microorganisms: the efficacy of aPDT against MDR microbial strains is similar to that of sensible ones. This efficacy is independent of the spectrum of resistance to conventional antimicrobials by the pathogen. (4). Rapid lethal effects: although conventional treatments take hours or even days and repeated doses to induce effects, PDT exhibits a rapid killing effect. It is estimated that a single PS molecule can generate 10,000 molecules of singlet oxygen. (5). Safety and nontoxicity: at the normally used concentrations in aPDT (µM range), photo antimicrobials are harmless to the tissues, either excited by light or not. They inactivate effectively the microorganisms at very low concentrations. (6). This method is easy to implement. This therapy only requires a light source and the presence of molecular oxygen and a suitable PS. (7). Low risk to induce mutagenic effects.

Although the broad spectrum of aPDT activity could be useful for empirical treatment, the lack of selectivity to microorganisms can be also regarded as a disadvantage when the treatment is applied to treat human infections. However, this drawback can be bypassed, by using delivery approaches to achieve the inactivation of the pathogenic microorganism without the compromise of the human microbiome. Different drug delivery systems have been tested for aPDT, such as antibodies and liposomes, and, more recently, new biomaterials, with promising results [88,107,108]. The advances in biotechnology allowed the development of new drug delivery systems of PS with superior therapeutic properties and less toxic effects and also encouraged the use of new materials. Biocompatible polymers are a good example of these new biomaterials, which have valuable biological properties [88].

The valid use of aPDT to control human microbial infections can be achieved using the free form of the PS. However, this approach is far from appropriate for application to disinfect medical devices, such as catheters and surfaces, where residual traces of PS would certainly be unacceptable. Free PS might not only introduce residual traces of sensitizer but would also make this technology more expensive. The immobilization of efficient PS in insoluble supports can be an interesting approach to inactivate pathogenic microorganisms present in surfaces. In fact, some research groups developed on solid supports with immobilized PS and tested their efficacy in inactivating different microorganisms [88,109]. Moreover, the immobilization of the PS on solid supports avoids its release with the environment, but also allows its recovery and readjustment, which makes the aPDT approach cost-effective and environmentally-friendly.

## 6. Blue Light Microbial Photoinactivation

### 6.1. Mechanism of Action of Blue Light

As mentioned before, aBLT is a specific type of light therapy. The principle of aBLT is the same as for aPDT, using a wavelength of visible light comprised between 400 to 470 nm, contrarily to conventional aPDT and dispenses the use of exogeneous PS [110]. Although the mechanism is not yet fully understood, the mostly accepted explanation is that the aBLT activates naturally occurring endogenous PS of pathogens, which leads to the formation of ROS. These ROS, as mentioned before for aPDT, through oxidation, result in cytotoxicity by reacting with proteins, lipids, and nucleic acids, of microbial cells, which leads to cell death [111,112]. The blue light inactivation effect on microorganisms is oxygen dependent. Thus, the increase of the quantity of oxygen, provides a superior action of ROS, which decreases the dose of light required to inactivate pathogens [113].

### 6.2. Endogenous Photosensitizers of Microorganisms

Although only a few studies are yet available regarding the topic of endogenous photosensitizers, it is known that aBLT exerts its actions mainly by iron-free porphyrins (Table 2). These iron-free porphyrins have two possible origins. They are either synthetized by bacteria as a by-product of heme biosynthesis or they arise as residuum of porphyrins that had their heme taken by the bacteria [114,115,116,117,118].

Studies demonstrated that the aBLT oxidation effect was due to the presence of coproporphyrin III and/or uroporphyrin III within *P. aeruginosa* cells [6,119]. The same research group also described the presence of protoporphyrin IX not only in *P. aeruginosa,* but also in *A. baumannii* [114]. As for *S. aureus* and *C. albicans*, uroporphyrin and coproporphyrin, and flavins, respectively, were the almost exclusively produced photo-sensitizers [117,118,120]. The inactivation of *H. pylori* was also found to be related with coproporphyrin and protoporphyrin IX [111,121].

### 6.3. Effectiveness of Blue Light in the Inactivation of Microorganisms 

An indispensable characteristic of a microorganism to be inactivated by aBLT is the presence of photosensitisers. Furthermore, recent studies demonstrated the presence of endogenous photosensitizing chromophores in several microbial strains, which are commonly found in hospital environments, such as *S. aureus*, MRSA, *P. aeruginosa*, *Klebsiella pneumoniae*, *Clostridium difficile*, *Streptococcus pyogenes*, *Mycobacterium spp*, *Salmonella, H. pylori, A. baumannii*, and *C. albicans* [6,111,112,114,118,122,123,124,125,126]. Notably, Gram-positive bacteria are supposed to be usually more sensible to aBLT than Gram-negative bacteria [7]. 

As mentioned before, one of the biggest flaws of antibiotics is the emergence of resistance after some time of use, which leads to failure to treat MDRO infections. Inversely, aBLT, similarly to aPDT, is effective against a wide variety of pathogens, regardless of their classic drug resistance profile [114,122,123,127,128,129]. Several researchers tried to understand the potential of the arising of resistance to aBL. Guffey et al. discovered that *S. aureus* can develop resistance if the light therapy is not correctly used [130]. However, it was found that resistance to aBLT is very unlikely to occur, which is similar to other light therapy modalities [129,131,132]. 

Numerous studies recognized the effectiveness of aBLT to inactivate pathogenic bacteria. Recently, Huang et al. were able to decrease MDR *E. coli* colony forming units (CFU) by 4–5 log10 [133]. Dai et al. inactivated 4.75 log10 CFU of MRSA [123]. Fila et al. successfully reduced *P. aeruginosa*, wild type and MDR, by 5.2 and 8 log10 CFU [134]. Halstead et al. demonstrated that all of the 34 different planktonic phase bacteria, specific tested in vitro, including *K. pneumoniae* and *E. faecium*, were susceptible to aBLT, with 71% of them suffering a ≥5 log10 CFU decrease after 15 to 30 minutes of exposure [135]. Bacterial biofilms also suffered significant decreases [135]. Wang et al. confirmed relevant antimicrobial activity of aBLT towards Gram-negative pathogens in biofilms, which reduced a 3.59 log10 CFU of *A. baumannii* biofilms and 3.02 log10 CFU of *P. aeruginosa* [114]. Moorhead et al. were able to inactivate *C. difficile* vegetative cells and spores by 3 log10 CFU, even though the inactivation of spores required 10 times more dose of light [136]. Zhang et al. inactivated 1.75 log10 CFU of *C. albicans* following a single exposure to light [118]. Wang et al. performed a review in 2017 about the capacity of aBLT to inactivate pathogens. They verified that more than 47 different pathogens, possible agents of HAIs, were successfully inactivated by aBLT [110].

Therefore, aBLT stands out as an effective method of disinfection against a great variety of major agents responsible for HAIs, even MDRO. Thus, the tremendous potential of aBLT for disinfection of hospital facilities is easily understandable, without the addition of any external agent, which is contrary to conventional aPDT light therapy. 

### 6.4. Advantages and Drawbacks of Antimicrobial Blue Light Relatively to aPDT 

As previously mentioned, light therapy modalities, such as aBLT and aPDT, exhibit a significant potential for the disinfection of hospital settings. Nevertheless, each one has its proper characteristics, advantages, and drawbacks (Table 2).

Unfortunately, aBLT is not a perfect technique. It also has its drawbacks. The rapid increase of ROS leads to its interaction with retinal photoreceptor cells, which causes oxidative stress and, consequently, severe eye damage. However, the use of eye-protectors and eye antioxidants can prevent oxidative damage [137,138,139].

### 6.5. High Intensity Narrow Spectrum Light

High Intensity Narrow Spectrum light (HINS-light) is a concretization of aBLT in the disinfection of healthcare settings, using an inactivating blue light with a wavelength of 405 nm, which was proven to be the most effective [126]. 

This approach, as previously mentioned, displays numerous advantages, which is an innovative procedure that would be useful for continuous irradiation in clinical areas, even in the presence of staff and patients, since the light used is harmless. This would provide an incessant control of environmental agents, even of MDROs, which is a fact that could substantially improve the actual paradigm of HAI control, sparing many human lives and considerable financial resources. 

Maclean et al. conducted several studies in this area. One of these studies demonstrated the in vitro effectiveness of a 405 nm LED light to inhibit several bacteria accountable for HAIs. HINS light was able to reduce Gram-positive species as *S. aureus*, MRSA, *S. epidermidis*, *C. perfringens*, and *S. pyogenes* by around 5 log10 CFU with a low light dose (around 40 J/cm^2^). Notably, *E. faecalis* was not susceptible to achieve a reduction of 2.6 log10 CFU. It required a higher light dose (216 J/cm^2^). Gram-negative bacteria were also less susceptible. Higher light doses were necessary (around 180 J/cm^2^) to reduce approximately 4 log10 CFU [126]. 

The same group conducted a study in a Scottish hospital, using a 2 HINS environmental decontamination system (HINS-light EDS) placed in an isolation room. First, it was tested empty. The HINS-light EDS working for 24 h reduced in 92% *S. aureus* contamination levels. Secondly, the system was tested while a patient with MRSA was admitted and the levels of the pathogen were reduced by 65%. Lastly, a room was occupied by a patient with MRSA and the MRSA concentration was determined before, during, and after the use of HINS-light. With the use of HINS-light, MRSA levels decreased by 50%. However, shortly after the light being turned off, *S. aureus* levels recovered by 98%, which corroborates the need of a continuous treatment to effectively reduce the environmental burden [140]. Other studies demonstrated that this technology is also efficient to disinfect areas frequented by outpatients as well as intensive care units [141,142]. 

In fact, a diversity of other species showed to be susceptible to HINS-light, namely *L. monocytogenes* [143], *C. difficile* [136,144], *P. aeruginosa* [134], *A. baumannii*, *K. pneumoniae*, *P. vulgaris*, *E. coli*, *S. enteriditis*, *S. sonnei*, *Serratia* spp., *Aspergillus niger*, *C. albicans*, and *Saccharomyces cerevisiae* [145].

As previously mentioned, aBLT and, in particular HINS, exhibits a wide range of other potential applications, such as the very effective disinfection of orthopedic osteosynthetic biomaterials [146].

Since HINS-light EDS integrates the spectrum of aBLT, it shares its advantages, which include the continuous disinfection of air and surface treatment, the effectiveness against a wide variety of pathogens, few installing and maintenance requirements, no need for staff training, no compliance problems with staff and patients, and low financial costs [145].

Since such a therapeutic approach is recent, the number of studies performed to evaluate its effectiveness and applicability *in vivo* is still very limited. Therefore, more field work will be required to better understand the particularities of this therapy.

## 7. Conclusions

HAIs are a serious threat to our modern healthcare systems, carrying not only huge morbidity and mortality, but also tremendous financial costs. Further worsening the panorama, MDROs are increasing in hospital facilities, which is allied to the economical disinterest of pharmaceutical companies in producing new antimicrobials. This also diminishes the capacity of conventional antimicrobials to treat HAIs. Therefore, new options are required, especially in the context of HAIs, to avoid the total incapacity of treating MDROs, which would be a catastrophe, since even the most banal infection could lead to death due to the absence of a valid antimicrobial therapy.

A crucial pillar of action against HAIs should be its prevention with effective surveillance programs and ideally continuous disinfection of hospital settings. Unfortunately, current conventional methods, such as manual cleaning with detergents, are usually incapable of doing so. 

Hospital surface colonization constitute the reservoir for the maintenance and propagation of HAIs. As mentioned before, current methods lack effectiveness in eliminating pathogens. It is mandatory to counteract this trend. Fortunately, blue light therapy modalities constitute a relevant and continuously acting solution to this problem.

Thus, blue light therapy modalities represent promising approaches in the combat of HAIs. They are capable of effectively eliminating even the most dangerous MDROs without significant adverse effects to patients and materials, and the development of photo resistance. HINS-light EDS may be effectively used for the continuous control of colonization/infection in hospital settings.

Therefore, it is imperative to continue to explore these new promising techniques in order to easily transpose its application to the hospital facilities, including day-care centers and nursing homes.

## Figures and Tables

**Table 1:** Photodynamic inactivation process. Equations modified from Reference [41].

Equation	No.
SubstractH_2_ + PS → PSH• + SubstractH•	(1)
PS* + Substract → PS•^−^ + Substract•^+^ ou PS* + Substract → PS•^+^ + Substract•^−^	(2)
PS•^−^ + ^3^O_2_ → PS + O_2_•^−^	(3)
O_2_•^−^ + H^+^ ⇋ HOO•	(4)
2 HOO• → H_2_O_2_ + O_2_	(5)
H_2_O_2_ + Fe^2+^ → HO• + OH^−^ + Fe^3+^	(6)
BiomoleculeH + HO• → Biomolecule• + H_2_O	(7)
Biomolecule• + ^3^O_2_ → Biomolecule-OO• → products	(8)
PS* + ^3^O_2_ → PS + ^1^O_2_	(9)
Biomolecules + ^1^O_2_ → oxidative products	(10)

**Table 2:** Light therapies comparison.

	aBLT	aPDT
Requirement of exogenous photosensitizers	Not required [7]	Required [7]
Damage of self-cells	Reduced, risk of eye damage [137]	Negligible [53]
Resistance development	Improvable [107,108,109]	Improvable [107,108,109]
Effectiveness	High, even against MDRO [138]	High, even against MDRO [138,139]
Multitarget capacity	Yes, lipids, proteins, and nucleic acids [104,105,106]	Yes, lipids, proteins, and nucleic acids [104,105,106]
Response time	Quick lethal effects [104]	Quick lethal effects [104]
